# Supplementary material for: Cloud BioLinux: pre-configured and on-demand bioinformatics computing for the genomics community
Source: BMC Bioinformatics. 2012 Mar 19;13:42. doi: 10.1186/1471-2105-13-42 (PMC3372431; doi:10.1186/1471-2105-13-42)
Supplement: Additional file 1 — Supplementary 1 Cloud BioLinux software documentation in the form of a mini, self-contained website. Users need to download and uncompress the .zip file, and open through a web browser the "index.html" file available on the main directory. (ZIP 1823 kb). [file 1471-2105-13-42-S1.ZIP › Cloud-BioLinux-Package-Documentation/docs/clustalx.html]

Bio-Linux Software Documentation Pages

Back to search form

## clustalx

|  |  |
| --- | --- |
| Name | clustalx |
| Description | **Clustal X** is a windows interface for the ClustalW multiple sequence alignment program.  It provides an integrated environment for performing multiple sequence and profile alignments and analysing the results. The sequence alignment is displayed in a window on the screen. A versatile coloring scheme has been incorporated allowing you to highlight conserved features in the alignment. The pull-down menus at the top of the window allow you to select all the options required for traditional multiple sequence and profile alignment.  You can cut-and-paste sequences to change the order of the alignment; you can select a subset of sequences to be aligned; you can select a sub-range of the alignment to be realigned and inserted back into the original alignment.  Alignment quality analysis can be performed and low-scoring segments or exceptional residues can be highlighted.  **References:**  Thompson,J.D., Gibson,T.J., Plewniak,F., Jeanmougin,F. and Higgins,D.G. (1997) The ClustalX windows interface: flexible strategies for multiple sequence alignment aided by quality analysis tools. Nucleic Acids Research, 24:4876-4882. |
| Homepage | ftp://ftp-igbmc.u-strasbg.fr/pub/ClustalX/ |
| Remote Documentation | http://www-igbmc.u-strasbg.fr/BioInfo/ClustalX/ |
